# Flexible electronics for cardiovascular monitoring on complex physiological skins

**DOI:** 10.1016/j.isci.2024.110707

**Published:** 2024-08-12

**Authors:** Tianqi Zhang, Yunshen Wang, Xingdong Feng, Yizhou Zuo, Hannong Yu, Hong Bao, Fan Jiang, Shan Jiang

**Affiliations:** 1 Hangzhou Institute of Technology, Xidian University, Hangzhou 311200, China; 2 Department of Pneumology, Tianjin Children’s Hospital, Children’s Hospital, Tianjin University, Tianjin 300204, China; 3 Geriatric Medical Center, Hainan General Hospital, Hainan Affiliated Hospital of Hainan Medical University, Haikou 570311, China; 4 State Key Laboratory of Electromechanical Integrated Manufacturing of High-performance Electronic Equipments, Xidian University, Xi’an 710071, China

**Keywords:** Health sciences, Physics, Engineering

## Abstract

Cardiovascular diseases (CVDs) pose a significant global health threat, responsible for a considerable portion of worldwide mortality. Flexible electronics enable continuous, noninvasive, real-time, and portable monitoring, providing an ideal platform for personalized healthcare. Nevertheless, challenges persist in sustaining stable adherence across diverse and intricate skin environments, hindering further advancement toward clinical applications. Strategies such as structural design and chemical modification can significantly enhance the environmental adaptability and monitoring performance of flexible electronics. This review delineates processing techniques, including structural design and chemical modification, to mitigate signal interference from sebaceous skin, motion artifacts from the skin in motion, and infection risks from fragile skin, thereby enabling the accurate monitoring of key cardiovascular indicators in complex physiological environments. Moreover, it delves into the potential for the strategic development and improvement of flexible electronics to ensure their alignment with complex physiological environment requirements, facilitating their transition to clinical applications.

## Graphical abstract

**Figure:**
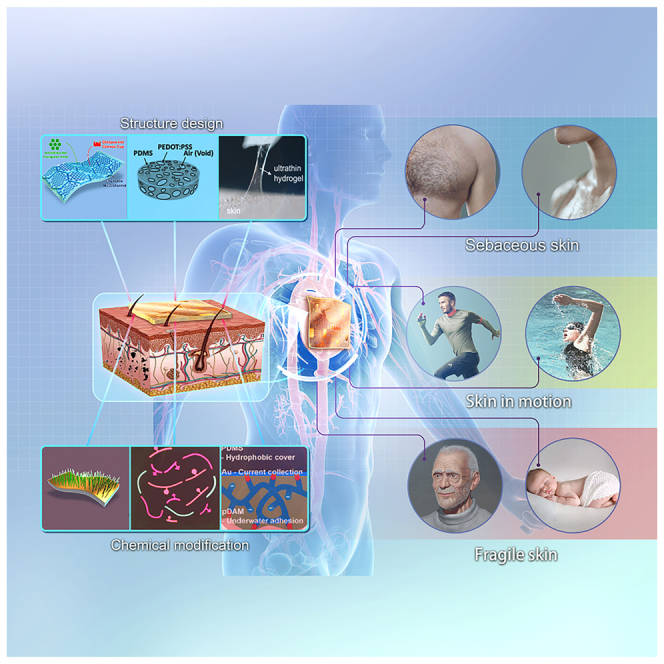

## Introduction

Cardiovascular diseases (CVDs) remain the leading cause of mortality worldwide, accounting for approximately 17.9 million deaths annually.^1^^,^^2^^,^^3^^,^^4^ These conditions, which encompass coronary artery disease, heart failure, stroke, and hypertension, pose significant public health challenges due to their chronic nature and the substantial burden they place on healthcare systems. Effective monitoring of cardiovascular health is crucial for early detection, timely intervention, and the prevention of adverse outcomes.^5^^,^^6^ Traditional surveillance methods, such as periodic clinical visits, blood pressure (BP) monitoring, electrocardiography (ECG), and echocardiography, have been the cornerstone of CVD management. BP measurement primarily monitors systolic and diastolic pressures. ECG mainly assesses heart rate, heart rhythm, various ECG wave segments, intervals, and ST segment changes to evaluate cardiac electrical conduction and detect myocardial ischemia or infarction. Echocardiography evaluates cardiac structure, function, blood flow velocity, and myocardial motion to assess overall heart health. Combined, these methods provide a comprehensive evaluation and early detection of cardiovascular diseases. However, these methods have notable limitations, such as their intermittent nature, reliance on patient compliance, and inability to provide continuous, real-time data.^7^^,^^8^

In recent years, advancements in flexible electronics have revolutionized the monitoring of CVDs.^9^^,^^10^^,^^11^^,^^12^^,^^13^^,^^14^^,^^15^^,^^16^^,^^17^^,^^18^^,^^19^^,^^20^^,^^21^^,^^22^^,^^23^^,^^24^^,^^25^^,^^26^^,^^27^^,^^28^^,^^29^^,^^30^^,^^31^^,^^32^^,^^33^^,^^34^^,^^35^^,^^36^ Flexible electronics, with their thin, soft, and wearable characteristics, can closely adhere to human skin, enabling continuous, real-time monitoring of physiological signals such as BP, pulse, ECG, and echocardiography.^37^^,^^38^^,^^39^^,^^40^^,^^41^^,^^42^^,^^43^^,^^44^^,^^45^^,^^46^^,^^47^^,^^48^^,^^49^^,^^50^^,^^51^^,^^52^^,^^53^^,^^54^^,^^55^ Real-time monitoring of physiological data is analyzed through an AI-assisted diagnostic platform, facilitating cardiovascular condition assessment. Compared to traditional devices, flexible electronics exhibit significant advantages in enhancing monitoring accuracy, portability, and patient comfort.^56^^,^^57^^,^^58^^,^^59^^,^^60^^,^^61^^,^^62^^,^^63^^,^^64^^,^^65^^,^^66^^,^^67^^,^^68^^,^^69^^,^^70^ However, despite significant progress in laboratory investigation, the application of flexible electronics in clinical medicine still faces numerous challenges. One of the challenges is ensuring the stable adhesion of devices under complex physiological environments.^71^^,^^72^^,^^73^^,^^74^^,^^75^^,^^76^^,^^77^^,^^78^^,^^79^^,^^80^^,^^81^^,^^82^^,^^83^

**Figure 1:**
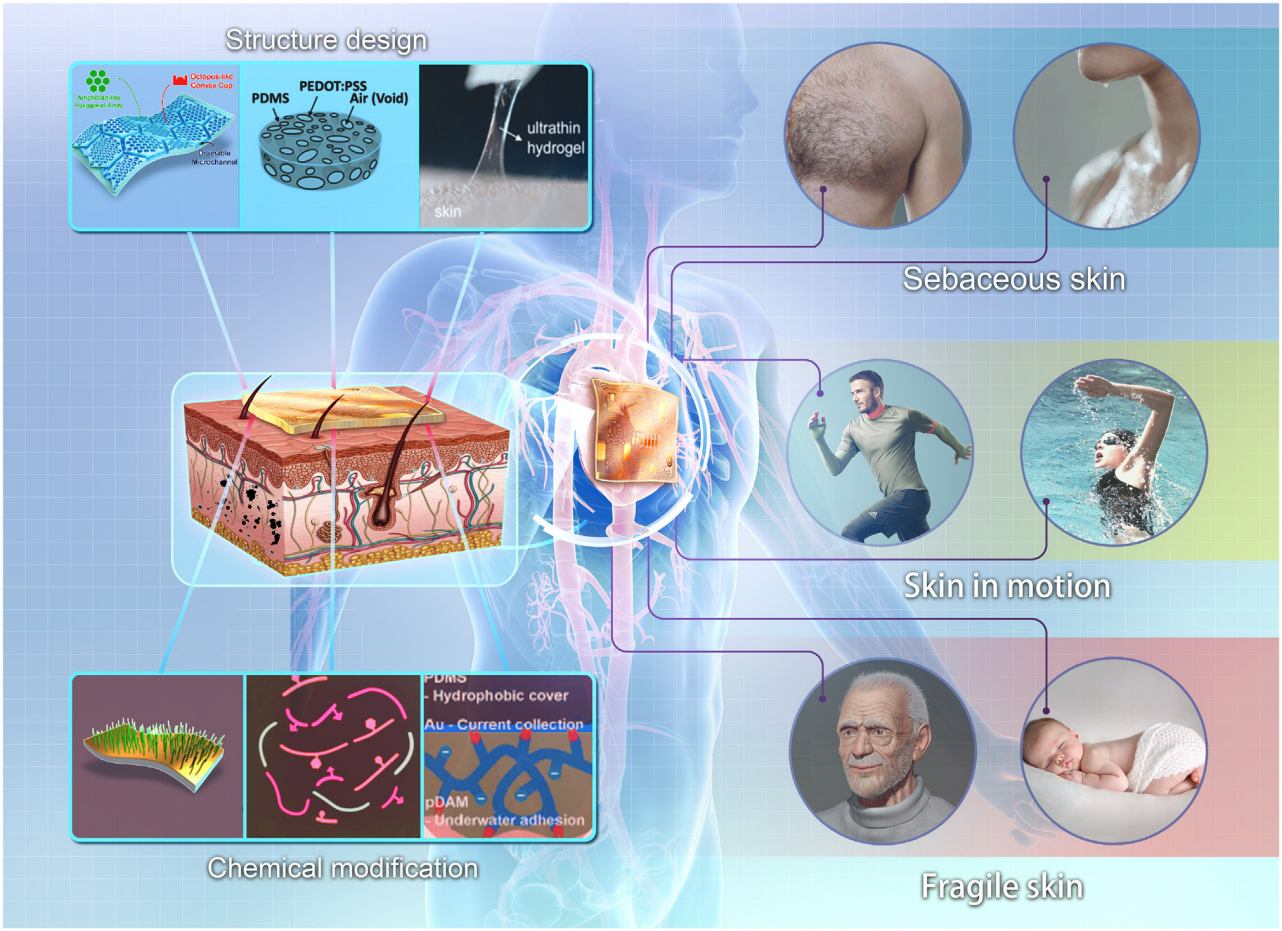
Flexible electronics for CVD monitoring in complex physiological environments

To address the challenge, strategies such as the optimization of structural design and surface chemical modification are studied by researchers, which can significantly enhance the environmental adaptability and monitoring performance of flexible electronics.^84^^,^^85^^,^^86^^,^^87^^,^^88^^,^^89^^,^^90^^,^^91^^,^^92^ Advancements in structural design boost the stability of device-skin contact, thereby enhancing signal acquisition accuracy. Meanwhile, chemical modification techniques can effectively improve the biocompatibility and environmental resistance of the devices, ensuring reliable operation under various harsh conditions. By now, existing reviews have predominantly concentrated on conceptual innovations or the novel functionalities of these devices, with scant attention given to the flexible electronics designs tailored for complex physiological environments.^9^^,^^61^^,^^93^^,^^94^^,^^95^^,^^96^^,^^97^^,^^98^^,^^99^ In this review, we delineate the methodologies employed in the design and fabrication of flexible electronics, including innovative structural designs and chemical alterations (Figure 1), specifically designed for the monitoring of CVDs within complex physiological environments. These devices are capable of addressing numerous clinical application problems, including signal interference from sebaceous skin, motion artifacts from moving skin, and infection risks associated with fragile skin. We also discuss how flexible electronics can be improved to align with complex physiological environments, thereby aiding the development of their clinical applications.

### Flexible electronics on various skins

The collection of physiological signals such as BP, pulse, ECG, and echocardiograms typically occurs near regions such as the wrist, neck, chest, and ears.^100^^,^^101^^,^^102^^,^^103^^,^^104^^,^^105^^,^^106^^,^^107^^,^^108^^,^^109^^,^^110^^,^^111^^,^^112^^,^^113^^,^^114^^,^^115^^,^^116^^,^^117^^,^^118^ Beneath the skin, there are usually numerous hair follicles, sebaceous glands, and sweat glands, which can lead to thick hair, oil, and sweat on the skin.^119^^,^^120^^,^^121^^,^^122^^,^^123^ These disturbances may cause faulty signal acquisition, potentially leading to misjudgments about cardiovascular conditions. Specifically, thick hair may interfere with the efficient interfacing of dry electrodes with the skin, significantly impacting the accuracy of long-term ECG signal acquisition. This problem also exists in wet electrodes, although it has been alleviated to a certain extent, but the signal stability still needs to be improved.^124^ For patients with oily skin, the electrode surface is prone to contamination by oils, as the materials typically used in flexible electronics—such as gold (Au), silver (Ag), and polydimethylsiloxane (PDMS)—are inherently oleophilic.^74^ The contamination may cause a rise in the skin-electrode impedance and degradation of signal quality due to the formation of a nonconductive electrical barrier and the bad contact caused by the thick oil layer.^125^ Furthermore, prolonged wear of these devices can lead to chronic skin rashes and damage due to metabolic blockage and limited biocompatibility. When the skin becomes sweaty, the unavoidable build-up of perspiration on the skin’s electronic interface can degrade the signal and cause the device to detach from the skin, thereby significantly compromising the accuracy of the gathered data. Additionally, if sweat is not adequately removed, it can cause discomfort and potentially lead to chronic respiratory skin obstruction, resulting in skin shield dysfunction (such as erythema) or internal conditions (such as allergies).^124^

Aerobic exercises such as jogging, yoga, and swimming can effectively enhance the cardiovascular function of patients and improve blood supply, but excessive exercise intensity will damage the cardiovascular system.^126^^,^^127^^,^^128^^,^^129^ Monitoring relevant physiological signals during exercise can effectively guide patients' exercise behaviors. However, when measuring ECG and other signals from moving patients using wet or dry electrodes, motion artifacts are likely to occur, leading to a weakened signal-to-noise ratio (SNR).^130^^,^^131^ The primary reason is that unstable contact between the electrodes and the skin can cause resistance changes, while the sliding of electrodes may lead to baseline drift and noise. Additionally, electrical signals generated by muscles during movement can interfere with the target signal. When the monitoring takes place under water or in high humidity environments, the acquisition of accurate signals becomes more difficult.^132^ Gel electrodes compromise their adhesion when exposed to water. Even when applied to the skin prior to immersion, water infiltrating the microvoids at the electrode-skin interface causes a swift degradation of electrode functionality. Although more viscous gel electrodes can function momentarily underwater, their higher impedance and lower stability result in significantly poor signal quality. For the acquisition of stable and reliable signals underwater, it is essential that gel electrodes maintain both conductivity and adherence to the skin even when submerged.

**Figure 2:**
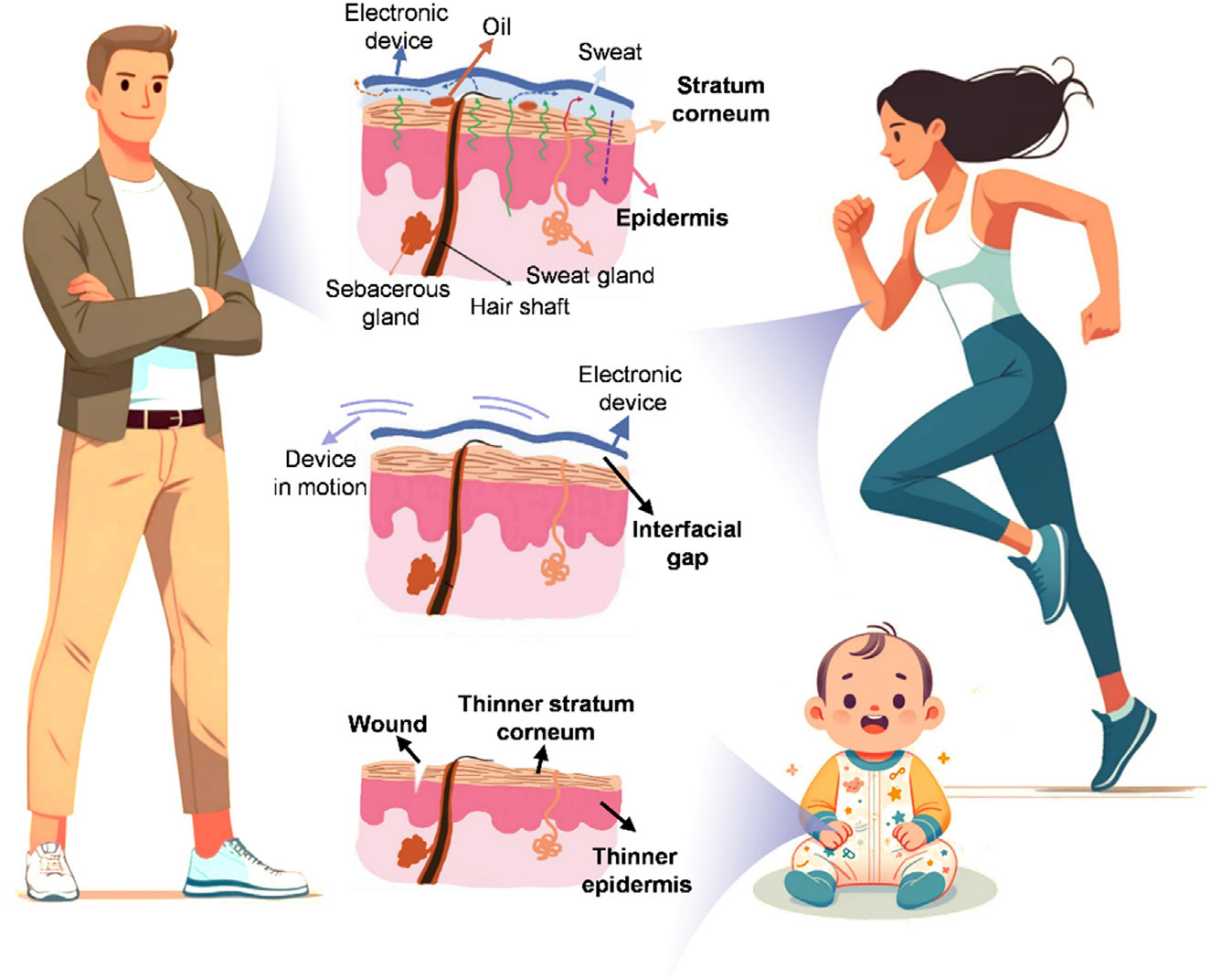
Adults and infant skin

Fragile skins also have a strong need for CVD monitoring such as injured patients, infants, and seriously ill patients.^133^^,^^134^^,^^135^^,^^136^ Contact between wounded skin and conventional electrodes heighten the risk of infection and irritation, and delays wound healing due to continuous pressure and friction.^137^^,^^138^^,^^139^ Additionally, compromised skin can hinder perfect contact between the skin and electrodes, reducing the SNR, while the inflammatory response can also distort the electrophysiological signal.^140^ The skin of infants and young children shares notable vulnerabilities with that of seriously ill patients, being exceedingly delicate (Figure 2). These individuals are susceptible to inflammation and allergic reactions when utilizing flexible electronics, such as heart rate and BP monitors. Particularly in neonatal and pediatric intensive care units, injuries from the removal of medical adhesives are a frequent concern.

### Flexible electronics for cardiovascular disease monitoring on sebaceous skin

Sebaceous skin is one of the most prevalent types of skin environment, where hair, oil, and sweat can directly interfere with the measurement of physiological signals by flexible electronics.^25^^,^^141^ Disturbances on sebaceous skin interfere with the efficient interfacing of the electrodes, potentially compromising the accuracy of long-term physiological signals acquisition. To better adapt to the complexities of the human epidermal environment, researchers have proposed strategies such as structure design and chemical modification to enhance the performance of electrodes.^81^^,^^142^^,^^143^^,^^144^^,^^145^

#### Structure design

**Figure 3:**
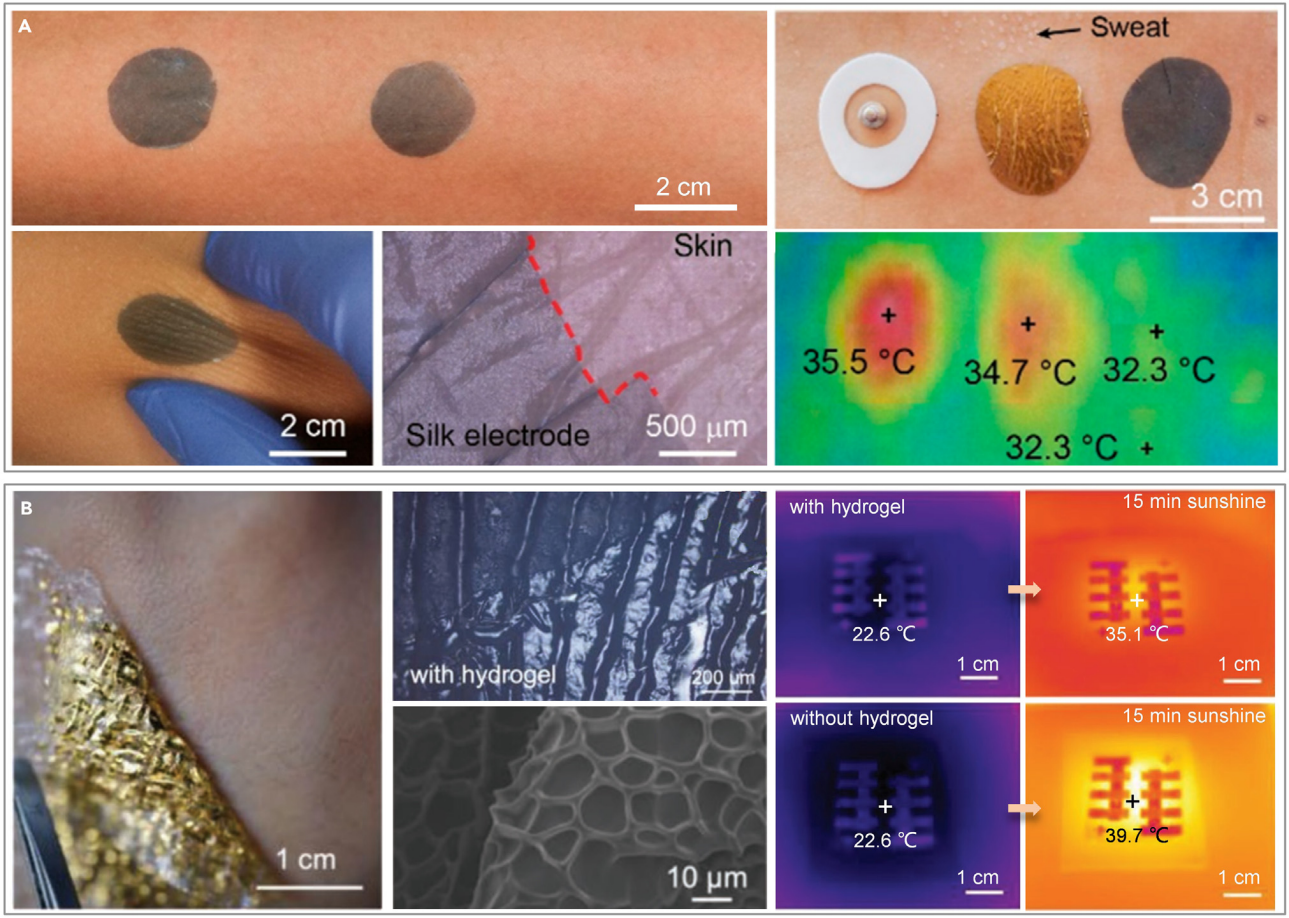
Structural design of flexible electronics for CVD monitoring on sebaceous skin (A) Fibrous electrode for sweaty skin. Reproduced with permission from ref. ^71^ Copyright 2021 American Chemical Society. (B) Ultra-thin hydrogel for sweaty skin. Reproduced with permission from ref. ^119^ Copyright 2022 Wiley-VCH.

The primary objective of the structural design is to diminish the instability inherent in the adhesion process, addressing concerns such as interface adhesion instigated by hair protrusion, signal quality deterioration due to the oil layer, and device instability provoked by sweat secretion. In terms of structural design, electrodes crafted from conductive fiber deposits are a common choice, as the fiber structure allows sweat to evaporate naturally, effectively mitigating the impact of sweat on physiological signal acquisition. Rogers et al. designed a filamentary serpentine gold electrode on microporous silicone.^146^ Chen et al. incorporated conducting polymers into glycerol-plasticized silk fiber mats (Figure 3A).^71^ Optical and infrared imagery of diverse skin electrodes, captured both pre- and post-exercise, accentuates the superior thermal conductivity of the silk-derived electrodes. Furthermore, the interface impedance between the moistened skin and electrodes diminishes. The electrodes exhibit a high water-vapor transmission rate (approximately 117 g m^−2^·h^−1^ under sweaty conditions, twice as high as the skin’s water loss). Although breathability is enhanced, perspiration removal between the skin and sensors still depends on natural evaporation rather than active absorption. To expedite sweat removal and adapt to hotter environments, Xu et al. developed a biomimetic e-skin composed of gold/thermoplastic polyurethane/cellulose membrane (Au/TPU/CM) that can immediately "pump" perspiration away from the interface through a combination of gradient porosity and surface energy gradient.^147^ This device boasts superior water vapor transmission and evaporation rates, 2.2 and 7.1 times greater than those of cotton fabric, respectively. Its ultrafast perspiration-wicking capability not only enhances comfort but also reduces the potential for measurement errors in skin hydration and temperature due to sweat, and eliminates the risk of short circuits in the sensor array.

In addition to fibrous electrodes, the researchers also proposed to add a layer of gel between the electrode and the skin to improve the interface environment between the skin and the electrode, which is conducive to the long-term monitoring of physiological signals. Wang et al. developed an on-skin paintable conductive biogel capable of a temperature-controlled reversible fluid-gel transition.^72^ This phase transition grants the biogel unique paintability directly on the skin and *in situ* gelatinization, enabling it to form conformal contact and maintain dynamic compliance with the contours of a hairy scalp. Demonstrated as an effective interface, this biogel facilitates long-term, high-quality EEG recordings spanning several days. In the premise of maintaining signal fidelity for a long time, enhance the skin’s respiration to reduce the probability of skin inflammation. An ultra-thin hydrogel is reported that conforms seamlessly to the glyphic lines and subtle minutiae on the skin, preventing the formation of air gaps (Figure 3B).^119^ The porous structure of the hydrogel promotes high water-vapor permeability, enabling nearly unrestricted transepidermal water loss and allowing the skin beneath to breathe freely. The corresponding infrared maps of the hydrogel interface, taken before and after 15 min of continuous illumination, revealed excellent thermal conductivity. Featuring exceptional mechanical compliance, high permeability, and biocompatibility, this ultrathin hydrogel interface enhances the widespread usability of skin-integrated electronics for extended periods, with proven long-term stability exceeding several weeks.

#### Chemical modification

**Figure 4:**
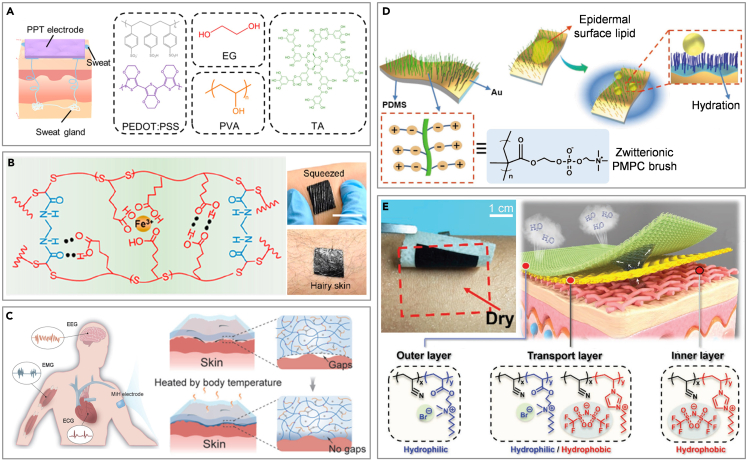
Chemical modification of flexible electronics for CVD monitoring on sebaceous skin (A) Intrinsic adhesive gel for sweaty skin. Reproduced with permission from ref. ^149^ Copyright 2022 American Chemical Society. (B) Supramolecular polymer for hairy skin. Reproduced with permission from ref. ^151^ Copyright 2022 American Chemical Society. (C) Thermal crosslinked adhesive gel for hairy skin. Reproduced with permission from ref. ^152^ Copyright 2022 Wiley-VCH. (D) Chemically grafted dry electrode for oily skin. Reproduced with permission from ref. ^74^ Copyright 2020 Wiley-VCH. (E) Chemically grafted fabric electrodes for sweaty skin. Reproduced with permission from ref. ^153^ Copyright 2021 Wiley-VCH.

Similar to the structural design, chemical modifications to the electrodes can also achieve better adherence to the regular human skin. Zhang et al. have developed an intrinsically self-adhesive electrode using a fully organic blend of poly(3,4-ethylenedioxythiophene): polystyrenesulfonate (PEDOT: PSS), waterborne polyurethane (WPU), and D-sorbitol.^148^ These electrodes effectively detect high-quality biopotential signals, due to their inherent adhesiveness and ability to conform to the skin. However, their adhesion weakens significantly when exposed to sweat. To address this problem, dry electrodes are prepared by drop casting blends comprising PEDOT: PSS, poly(vinyl alcohol) (PVA), and tannic ahadcid (TA) (Figure 4A).^149^ These electrodes maintain strong adhesion to both dry and wet skin, with adhesion forces measuring 0.28 and 0.32 N cm^−1^, respectively. The TA’s hydroxyl groups and hydrophobic benzene ring enable robust bonding to skin, while the soft PVA matrix helps in energy dissipation.^150^ Similarly, a supramolecular polymer (SESA) rich in amino and carboxyl groups, along with a percolation network of *in situ* transferred carbon nanotubes, is used to create a dry epidermal patch (Figure 4B).^151^ The patches, which feature numerous functional groups (−COOH, C=O, and N−H) on their surface, exhibit strong physical adsorption to the outermost stratum corneum via hydrogen bond formation.

Additionally, the temperature-sensitive rheological properties of a non-covalent dynamic network involving small molecules of branched polyethylenimine (bPEI) are utilized in another gel electrode (Figure 4C).^152^ The blend of bPEI, PVA, and Ca^2+^ enhances adhesion over time, creating gapless contact facilitated by body heat. The coordination of Ca^2+^ with hydroxyl groups is beneficial to the enhancement of mechanical properties. Although the intrinsically adhesive material can adapt to the sebaceous skin to a certain extent, the signal quality is still seriously affected in an environment full of sweat and grease. The environmental adaptability of the electrode can be adjusted by surface chemical modification. Zwitterionic molecules of poly(2-methacryloyloxyethyl phosphorylcholine) (PMPC) have been grafted onto electrode surfaces to create a polymer brush with anti-epidermal-surface-lipid (Figure 4D).^74^ This modification leverages the underwater oil-clearing properties of zwitterions, enabling the electrodes to be used on oily skin and cleaned with water alone. Significantly, electrodes contaminated by epidermal surface lipids can be effectively cleaned using pure water, thereby restoring high signal quality. Zheng et al. explored the integration of structural design and chemical modifications by altering the cation and anion components of a poly(ionic liquid) (PIL) and utilizing a spinning process (Figure 4E).^153^ This approach resulted in the development of a PIL-based multilayer nanofiber membrane electronic skin with dual gradients. The separation of positive and negative charges along the main chain contributes to the high conductivity, while the presence of quaternary ammonium ions on the side chains enhances the strong hydrophilicity and antimicrobial properties. Additionally, the pore size gradients in the PIL electronic skin enhance breathability and facilitate the acquisition of bioelectrical signals from the human body. Presently, the interface environment between electrodes and sebaceous skin can be effectively improved through structural design and chemical modification. The chemical grafting of the device enhances its adaptability to sebaceous skin, while its structural design ensures prolonged signal stability during extended monitoring periods. Both strategies possess distinct advantages and disadvantages. Furthermore, the amalgamation of these dual strategies in flexible electronics can markedly diminish the impact of surface impurities on the signal. Many challenges remain, including the long-term monitoring of signal accuracy and comfort and the impact of ambient temperature changes on the accuracy of signal collection.

### Flexible electronics for cardiovascular disease monitoring on the skin in motion

Studies have shown that physically active individuals have a 50% lower risk of CVDs than sedentary persons. The monitoring of BP, pulse, and ECG during activities aids in assessing cardiovascular conditions,^154^^,^^155^ which is helpful for the prevention and diagnosis of CVDs. For dry electrodes, the mismatch between the electrode and human skin may occur during sports, leading to serious motion artifacts. When the activity transpires underwater, the dry electrode becomes essentially impractical, whereas the wet electrode can momentarily gather physiological signals. When utilized underwater, the wet electrode renders the device inoperative as it tends to detach.

#### Structure design

**Figure 5:**
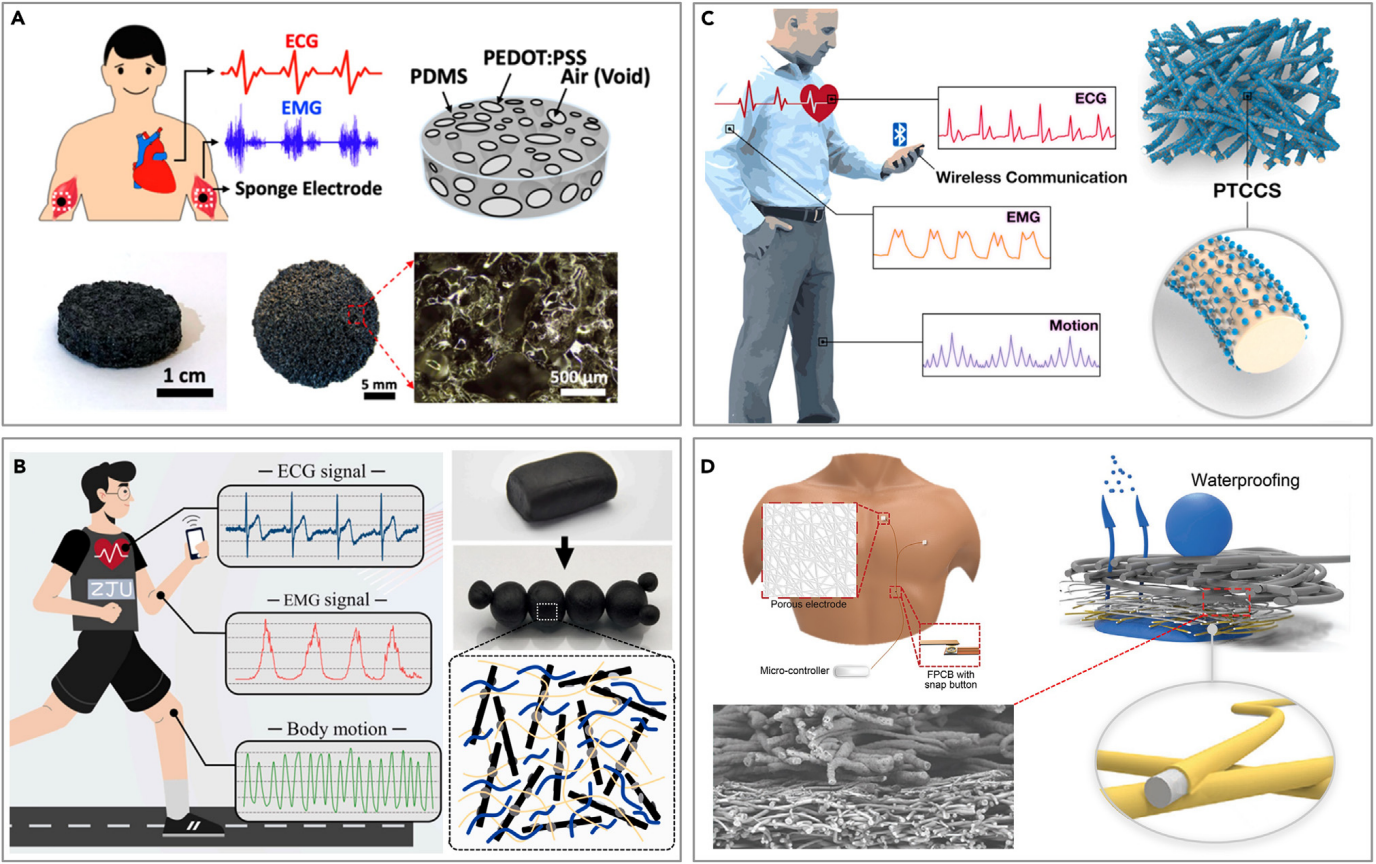
Structural design of flexible electronics for CVD monitoring on skin in motion (A) Sponge electrode for skin in motion. Reproduced with permission from ref. ^75^ Copyright 2022 American Chemical Society. (B) Nanocomposite electrode for skin in motion. Reproduced with permission from ref. ^85^ Copyright 2023 Elsevier. (C) Fiber electrode for skin in motion. Reproduced with permission from ref. ^158^ Copyright 2022 Elsevier. (D) Multilayer fiber electrode for skin in motion. Reproduced with permission from ref. ^159^ Copyright 2022 Wiley-VCH.

Dry electrodes are highly susceptible to motion artifacts, rendering them less ideal for biopotential monitoring due to sensitivity to body movement. To address these challenges, extensive efforts have been directed toward developing lightweight, skin-conformable soft electrodes for physiological signal recording. A notable strategy involves the use of microneedles that penetrate the nonconductive stratum corneum layer, thereby reducing electrode-skin contact impedance. For instance, electrodes with a Miura-Ori tessellation structure, made from a soft PDMS substrate and coated with a titanium-gold layer, can flex to maintain stable skin contact post-penetration.^156^ However, despite their benefits, the invasive nature of microneedles has sparked safety concerns and debates, particularly regarding potential biosafety risks if broken microneedles leave residues that could lead to skin redness and irritation.^157^ Additionally, Lo et al. have developed a soft sponge electrode, consisting of a porous PDMS sponge coated with a conductive PEDOT: PSS layer (Figure 5A).^75^ The porous structure of sponge electrodes significantly enhances the skin-electrode contact area, reducing impedance and consequently improving the SNR for biopotential signal recording. These electrodes also hold a greater amount of conductive hydrogel in their micropores, which helps reduce motion artifacts. The highly malleable conductive polymer, PEDOT: PSS, is also combined with Laponite-modified carbon nanotubes (CNTs) and poly(ethylene oxide) (PEO) to create a multifunctional, silly putty-like nanocomposite (LPPC) (Figure 5B).^85^ The nanocomposite electrode exhibits exceptional electrical conductivity, superb moldability to enhance contact area, and possesses the capability to record ECG signals seamlessly during movement.

In addition, textiles are adept at recording physiological signals from the moving human body and can accommodate body-motion-induced complex deformations.^160^ Furthermore, a nonwoven textile-based bioelectrode featuring a robust self-cleaning capability and ultra-high stretchability is developed. This electrode incorporates a synergistic combination of stretchable carbon black nanoparticle/CNT conductive networks and a superhydrophobic perfluorooctyltriethoxysilane-modified TiO_2_ nanoparticle surface for ECG signals collection (Figure 5C).^158^ The employment of superhydrophobic surfaces is recognized as an effective strategy for enhancing anti-fouling and anti-corrosion properties of sensing materials.^161^ Oh et al. proposed a hybrid structure of the hydro-wetting nanofiber mat electrode (Figure 5D).^159^ The complex three-layer structure consists of gold-coated block copolymer fibers, hydrophobic porous nanofiber mats, and 4 μm thick SEBS fiber gaskets. The Au electrode enables rapid sweat evaporation, maintaining uniform skin contact and preventing irritation. The hydrophobic mats render the outer surface of the electrode waterproof.

#### Chemical grafting

**Figure 6:**
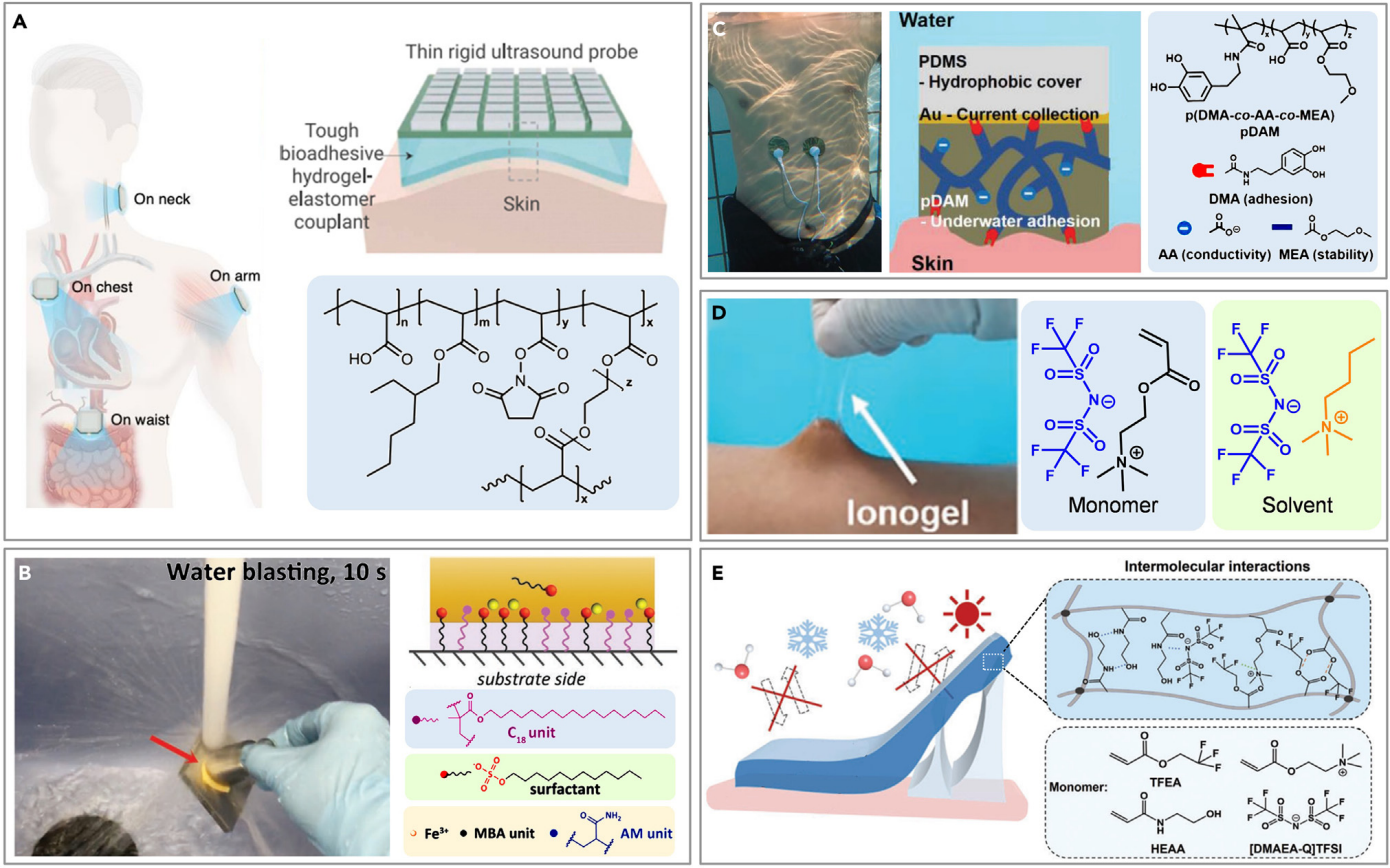
Chemical modification of flexible electronics for CVD monitoring on skin in motion (A) Intrinsic adhesive gel for skin underwater. Reproduced with permission from ref. ^162^ Copyright 2022 The American Association for the Advancement of Science. (B) Crosslinked copolymer hydrophobic hydrogel for skin underwater. Reproduced with permission from ref. ^163^ Copyright 2019 Wiley-VCH. (C) Waterproof electrode for skin underwater. Reproduced with permission from ref. ^164^ Copyright 2020 Wiley-VCH. (D) Ionic gel for skin underwater. Reproduced with permission from ref. ^80^ Copyright 2021 Wiley-VCH. (E) Organo–ionic gel for skin underwater. Reproduced with permission from ref. ^165^ Copyright 2023 Wiley-VCH.

In addition to enhancing the interface between the electrode and the skin via structural design, a gel with superior adhesive properties can be formulated through chemical modifications to ensure intimate contact with the skin, enabling the precise acquisition of physiological signals during physical activity. Wang et al. unveiled a bioadhesive ultrasound (BAUS) device that can deliver continuous imaging (echocardiograms) for up to 48 h, capturing detailed visuals of internal organs such as blood vessels, muscles, the heart, the gastrointestinal tract, the diaphragm, and the lungs (Figure 6A).^162^ This device uses a soft yet tough hydrogel made of chitosan-polyacrylamide interpenetrating networks and water, encapsulated by a polyurethane membrane, to maintain hydration and ensure comfortable contact with the skin. Organohydrogels, however, are susceptible to leakage of organic solvents due to their hydrophilic nature. A class of dynamic hydrophobic hydrogels consist of a crosslinked copolymer of hydrophilic acrylamide and hydrophobic stearyl methacrylate (C_18_), sodium dodecyl sulfate (SDS), and Fe^3+^ ions that offer repeatable, long-term stable underwater adhesion to various substrates (Figure 6B).^163^ In the hydrogels, the covalent linkage made from MBAA is designed to provide a permanent polymer network while the hydrophobic alkyl chains of the C_18_ units aggregate in SDS micelles to form dynamic hydrophobic associations.

The additional hydrogel layer, while effectively improving underwater adhesion, increases the resistance of the sensing circuit.^166^ To diminish resistance and amplify signal strength, researchers have incorporated conductive materials into the gel, thereby enhancing its conductivity. Ji et al. developed water-resistant electrodes for underwater ECG monitoring by synthesizing a copolymer from dopamine methacrylamide (DMA), acrylic acid (AA), and methoxyethyl acrylate (MEA), which is then applied to Au/PDMS films to create pDAM/Au/PDMS electrodes (Figure 6C).^164^ The dopamine component, DMA, facilitates underwater adhesion, while AA provides ionic conductivity, essential for transmitting electrophysiological signals in water. Ionic liquids, renowned for their exceptional electrical conductivity, are also employed to augment the conductivity of gels. Yu et al. crafted an ionogel via a one-step free radical polymerization process using acryloyloxyethyltrimethylammonium bis(trifluoromethanesulfonyl)imide ([DMAEA-Q][TFSI]) in butyltrimethylammonium bis(trifluoromethanesulfonyl)imide ([N4111][TFSI]) (Figure 6D).^80^ Fluoropolymers, characterized by fluorine’s high electronegativity, demonstrate low surface energy, minimal moisture absorption, and excellent chemical resistance.^167^^,^^168^ These properties enable fluoropolymer-based ionogels to withstand water interference, exhibiting enhanced adhesion, conductivity, and stability underwater. They effectively collect real-time ECG signals both in air and underwater, alerting users to potential heart attack risks. Furthermore, a versatile, organo-ionic gel-based electrode (OIGE) has been developed, employing a synthesis of the highly conductive choline-based ionic liquid ([DMAEA-Q] [TFSI], I.L.) and select monomers - namely, 2,2,2-trifluoroethyl acrylate (TFEA) and N-hydroxyethyl acrylamide (HEAA) (Figure 6E).^165^ The TFEA contribute to a robust chain structure that imparts exceptional water resistance, adhesion, and electrical conductivity to the electrode. The OIGE demonstrates resistance to sweat and water, anti-freezing properties, and anti-dehydration qualities, maintaining formidable adhesiveness and electrical stability in diverse environmental conditions.

At present, the structural design makes the electrode light, soft, and breathable, and effectively increases the contact area between the electrode and the skin, which effectively reduces the impedance and ultimately weakens the purpose of motion artifacts. Besides, the gel between the electrode and skin can be modified to enhance the adhesion and conductivity underwater, achieving stable underwater adhesion and low-impedance signal recording. Currently, ionic liquids are commonly used to enhance the conductivity of gel electrodes, with priority given to those possessing high conductivity, low volatility, and low toxicity. The structural design of flexible electronics can adeptly mitigate motion artifacts in conventional movements, and the chemical modification of flexible electronics is crucial as well, primarily to address device damage caused by underwater motion. Both aspects have distinct priorities and are of paramount importance. The remaining challenges include the complete elimination of motion artifacts, long-term underwater monitoring, mechanical stability of electrodes in motion, and reliable operation at clinical-grade levels of accuracy.

### Flexible electronics for cardiovascular disease monitoring on fragile skin

For patients with CVDs who have undergone surgical treatment, ECG or BP monitoring is usually required for a longer period of time after surgery to avoid disease deterioration. Physiological signals monitoring of cardiovascular conditions in patients with injured skin and infantile patients is paramount to ensuring no opportunity for the diagnosis and treatment of the disease is missed.^169^ These patients have fragile skin, heightening their vulnerability to infection and irritation, and making them susceptible to injury from the high peeling force of the strongly adhesive electrode.

#### Structure design

**Figure 7:**
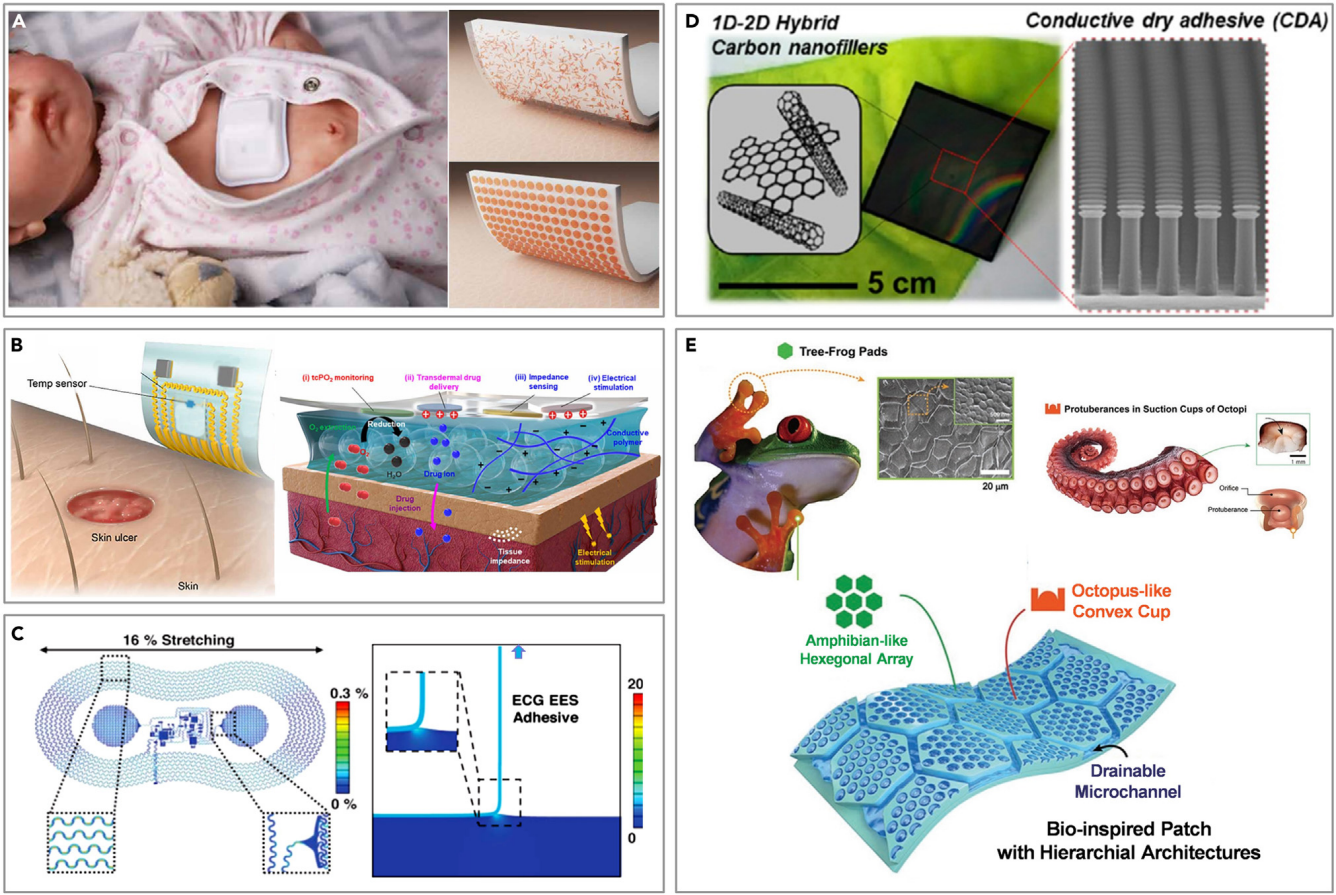
Structural design of flexible electronics for CVD monitoring on fragile skin (A) Thermally switchable composite silicone material for infant skin. Reproduced with permission from ref. ^82^ Copyright 2022 The American Association for the Advancement of Science. (B) Ultra-thin hydrogel for skin ulcer. Reproduced with permission from ref. ^172^ Copyright 2021 The American Association for the Advancement of Science. (C) Electrodes with microfluidic chamber for fragile skin. Reproduced with permission from ref. ^173^ Copyright 2019 The American Association for the Advancement of Science. (D) Adhesives with mushroom-shaped tips for fragile skin. Reproduced with permission from ref. ^175^ Copyright 2016 American Chemical Society. (E) Biomimetic electrode for fragile skin. Reproduced with permission from ref. ^84^ Copyright 2019 Wiley-VCH.

Accurate physiological signal collection is crucial for diagnosing CVDs, particularly in the neonatal intensive care unit (NICU) and intensive care unit (ICU). The electrodes are attached to the skin with strong adhesives that can irritate and cause iatrogenic injuries upon removal. An innovative approach involves a thermally switchable composite silicone material that allows for fast, wirelessly triggered reductions in adhesion (Figure 7A).^82^ The composite material blends crystallizable oil—consisting of fatty alcohols (1-pentadecanol and 1-hexadecanol) and linear alkanes (n-dodecane and eicosane)—with a melting temperature carefully selected to be slightly above the natural temperature range of various skin surface areas (33°C–37°C).^170^ This oil is distributed in a random crystalline form throughout the thickness of the silicone base or in a patterned circular disk form near the surface.^171^ Under typical conditions, the oil stays solid, ensuring the device’s firm adhesion to the skin. Upon mild heating, the oil transitions to a liquid state and diffuses through the silicone adhesive layer to the skin interface. Furthermore, the adhesion of hydrogels can be regulated by incorporating specialized structures, simultaneously facilitating molecular transport. Lim et al. developed a novel type of ultrathin, porous hydrogel, which they then functionalized and incorporated into wearable biosensors for enhanced signal quality and comfort (Figure 7B).^172^ Due to the presence of the ultra-thin hydrogel, the sensor can adhere to the site of the skin ulcer, subsequently allowing for mild heating by other devices. The porous structure of the hydrogel facilitates the uptake of oxygen from the blood vessels under the skin to measure changes in physiological signals, such as blood flow and local oxygen saturation level. Chung et al. developed an epidermal electronic system (EES), featuring a microfluidic chamber filled with a nontoxic ionic liquid (1-ethyl-3-methylimidazolium ethyl sulfate) positioned between the electronics and the lower encapsulation layer to ensure mechanical isolation (Figure 7C).^173^ The thin, soft mechanical properties of sensors allow them to adhere using only van der Waals forces, with adhesion energy determining the steady-state peeling force.^174^ These sensors feature effective moduli in the range of 200–300 kPa, minimizing the normal and shear stresses at the skin interface during natural neonate movements. The integration of the microfluidic channel further reduces these stresses by up to 2.5 times compared to similar designs lacking microfluidics. Additional stress reduction is possible through perforations in the platform’s open areas, which also lower the effective modulus of the EES, enhancing its deformability under force.

Innovative bionic structures represent a promising direction in design evolution. Various bioinspired skin adhesives with multiscale architectures have emerged, including patches with mushroom-shaped tips, microneedles, and clingfish-inspired microgrooves.^176^^,^^177^^,^^178^^,^^179^^,^^180^ Notably, adhesives with mushroom-shaped tips improve attachment via van der Waals forces on dry skin and maintain stable underwater contact when integrated into swimwear (Figure 7D).^175^ These designs ensure strong adherence while maintaining low peel forces, making them ideal for individuals with fragile skin. In addition, part of the study also carried out structural combination design on the basis of this, such as the hexagon structure of the amphibian foot and the convex cup structure in the octopus sucker (Figure 7E),^84^ which can realize the physiological signals collection of fragile skin in the humid environment.

#### Chemical grafting

**Figure 8:**
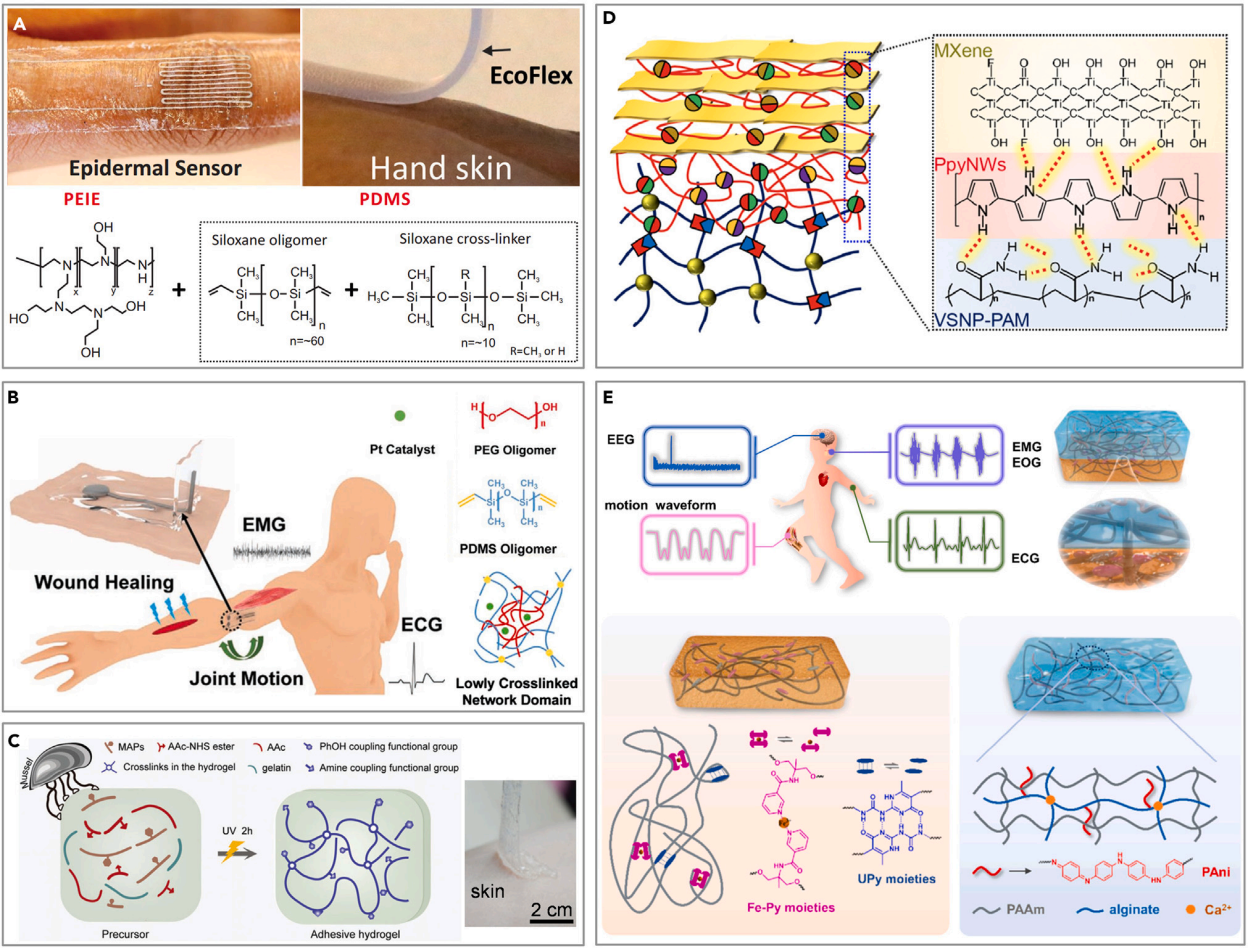
Chemical modification of flexible electronics for CVD monitoring on fragile skin (A) Low crosslinking PDMS with easy stripping property. Reproduced with permission from ref. ^182^ Copyright 2016 Wiley-VCH. (B) Low crosslinking PDMS for injured skin. Reproduced with permission from ref. ^183^ Copyright 2022 Wiley-VCH. (C) Gel composed of biomass for fragile skin. Reproduced with permission from ref. ^184^ Copyright 2023 Wiley-VCH. (D) Mixed hydrogel with biocompatibility. Reproduced with permission from ref. ^185^ Copyright 2020 The American Association for the Advancement of Science. (E) Biomimetic double-network hydrogel for baby skin. Reproduced with permission from ref. ^186^ Copyright 2022 Elsevier.

For individuals with delicate skin, the chemical composition of the skin-friendly polymer or gel can be adjusted to promote wound healing and reduce peeling damage. PDMS, renowned for its commendable biocompatibility, exhibits limitations in adhesiveness and conformability. Researchers reduce the crosslinking degree of PDMS by solvent-thermal curing,^181^ adding chemical reagents (Figure 8A)^182^ or reducing the amount of crosslinking agent, making it more suitable for fragile skin. Sylgard 184 is a PDMS elastomer composed of two components: double-ended vinyl dimethylsiloxane and hydrosiloxane. The incorporation of a specific quantity of PEG oligomers into its precursor can diminish crosslinking, resulting in a loosely cross-linked network (Figure 8B).^183^ The resulting PEG-PDMS adhesive displays exceptional softness, adhesiveness, and stretchability. Leveraging these properties, the electronics are applicable in areas such as motion detection, electrophysiology, and wound healing.^183^ To enhance biocompatibility, a novel adhesive hydrogel (AH) is created using a simple one-step method, integrating mussel adhesive proteins (MAPs) and gelatin from porcine skin, both rich in catechol groups (Figure 8C).^184^ The AH’s strong adhesion is driven by multiple interactions with the target surface, including hydrogen and covalent bonds, along with π–π and charge-charge interactions, facilitated by various polar groups such as catechol, carboxyl, and NHS ester groups. The adhesiveness of AH can be optimized by adjusting the MAP content, which influences shear and tensile strength by altering the balance between intramolecular interactions and surface adhesion. Unlike similar products, AH does not require prior polar treatment of the target surfaces and features easy-to-peel-off properties, enhancing efficiency in clinical application.

Polypyrrole nanowires (PpyNWs), recognized for their biocompatibility, establish a connection between the vinyl-hybrid-silica nanoparticle (VSNP)-modified polyacrylamide (PAM) hydrogel and the two-dimensional (2D) MXene, thereby constructing a versatile electronic-skin apparatus (Figure 8D).^185^ Following the incorporation of a highly biocompatible substance, the detrimental impact on the fragile skin is substantially mitigated. Similarly, the integration of optimal physical crosslinking mechanisms, such as hydrogen bonding and metal coordination, into the biocompatible polyurethane elastomer significantly enhances its flexibility and stretchability, as illustrated in Figure 8E.^186^ The dynamic hydrogen bonding of 2-ureido-4[1H]-pyrimidinone (UPy) units, along with the metal-ligand coordination of Fe^3+^-pyridine (Fe-Py) moieties, are strategically incorporated within the covalently cross-linked polyurethane (CPU) elastomeric polymer network (CPU-Fe-Py-U). Additionally, the hydrogel precursor containing polyaniline (PAni) is polymerized *in situ* on the elastomer surface, resulting in a hydrogel characterized by a double-polymer network, superior ductility, and exceptional electrochemical properties. The elastomer-hydrogel integration (EHI) demonstrates ultra-rapid responsiveness and remarkable resilience, serving effectively as a skin sensor capable of detecting a wide range of physiological signals with high sensitivity and precise signal waveform interpretation.

In the present study, structural design is decidedly prioritized to optimize the interface between fragile skin and devices, as the sensitive nature of delicate skin prohibits prolonged exposure to gels or other chemical substances. For longer monitoring, it is important to use skin-friendly polymers, such as silk fibroin, PEDOT, polyurethane, polyacrylate, PDMS, and natural biopolymers. The primary challenge lies in efficiently capturing biological signals while minimizing skin impact, with porous-structured electrodes offering significant advantages.

### Conclusion and outlook

Flexible electronics have significant advantages in the monitoring of CVDs in terms of monitoring accuracy, portability, and patient comfort. However, many of the research and device prototypes in this field are still in their infancy, mainly confined to the laboratory investigation stage, with little consideration of the complex physiological environments. Unlike many previous reviews on flexible electronics, which primarily focus on materials, functionality, and design within experimental settings, this article further elucidates the requirements for flexible electronic design for CVD monitoring in complex physiological environments. It highlights the importance of structural design and chemical modification. To enhance clinical applicability, structural design, and chemical modification must collaborate to enable the effective flexible electronic monitoring of CVDs in intricate physiological contexts. The structural design and chemical grafting of flexible electronics can synergistically address various external challenges, facilitating more intricate physiological environment monitoring, such as for sweaty infant skin and oily skin during exercise.

Several important branches of future work include wireless integration, miniaturization, intelligent healthcare, AI diagnostics, and advanced materials. The wireless capabilities and miniaturization of flexible electronics can reduce the data acquisition system to the size of a mobile phone, thereby enhancing the portability of detection equipment. Advanced manufacturing materials can fundamentally enhance the strength and accuracy of flexible electronic signal acquisition. When coupled with sophisticated artificial intelligence diagnostic systems, this can significantly reduce the misdiagnosis rate of cardiovascular diseases. Finally, flexible electronics represent a highly interdisciplinary field characterized by inherent complexity and diversity. The advancement and enhancement of these devices depend on collaboration among multidisciplinary researchers. The necessity for flexible electronics to precisely monitor CVDs in complex physiological environments underscores their potential for further exploration.
